# Systematic computational identification of prognostic cytogenetic markers in neuroblastoma

**DOI:** 10.1186/s12920-019-0620-6

**Published:** 2019-12-12

**Authors:** Chao Qin, Xiaoyan He, Yanding Zhao, Chun-Yip Tong, Kenneth Y. Zhu, Yongqi Sun, Chao Cheng

**Affiliations:** 1 Beijing Key Lab of Traffic Data Analysis and Mining, School of Computer and Information Technology, Beijing Jiaotong University, No.3 Shangyuancun, Beijing, 100044 Haidian District China; 2 Department of Medicine, Baylor College of Medicine, BCM451, Suite 100D, Houston, TX 77030 USA; 3 Center for Clinical Molecular Medicine, Children’s Hospital, Chongqing Medical University, Ministry of Education Key Laboratory of Child Development and Disorders, Key Laboratory of Pediatrics in Chongqing, Chongqing International Science and Technology Cooperation Center for Child Development and Disorders, Chongqing, 400014 China; 4 Department of Biomedical Data Science, Geisel School of Medicine at Dartmouth, Lebanon, NH 03766 USA; 5 Department of Biological Sciences, Dartmouth College, Hanover, NH 03755 USA

**Keywords:** Cytogenetic marker, Neuroblastoma, Chr11p14, Chr11q23, Prognosis

## Abstract

**Background:**

Neuroblastoma (NB) is the most common extracranial solid tumor found in children. The frequent gain/loss of many chromosome bands in tumor cells and absence of mutations found at diagnosis suggests that NB is a copy number-driven cancer. Despite the previous work, a systematic analysis that investigates the relationship between such frequent gain/loss of chromosome bands and patient prognosis has yet to be implemented.

**Methods:**

First, we analyzed two NB CNV datasets to select chromosomal bands with a high frequency of gain or loss. Second, we applied a computational approach to infer sample-specific CNVs for each chromosomal band selected in step 1 based on gene expression data. Third, we applied univariate Cox proportional hazards models to examine the association between the resulting inferred copy number values (iCNVs) and patient survival. Finally, we applied multivariate Cox proportional hazards models to select chromosomal bands that remained significantly associated with prognosis after adjusting for critical clinical variables, including age, stage, gender, and *MYCN* amplification status.

**Results:**

Here, we used a computational method to infer the copy number variations (CNVs) of sample-specific chromosome bands from NB patient gene expression profiles. The resulting inferred CNVs (iCNVs) were highly correlated with the experimentally determined CNVs, demonstrating CNVs can be accurately inferred from gene expression profiles. Using this iCNV metric, we identified 58 frequent gain/loss chromosome bands that were significantly associated with patient survival. Furthermore, we found that 7 chromosome bands were still significantly associated with patient survival even when clinical factors, such as *MYCN* status, were considered. Particularly, we found that the chromosome band chr11p14 has high potential as a novel candidate cytogenetic biomarker for clinical use.

**Conclusion:**

Our analysis resulted in a comprehensive list of prognostic chromosome bands supported by strong statistical evidence. In particular, the chr11p14 gain event provided additional prognostic value in addition to well-established clinical factors, including *MYCN* status, and thereby represents a novel candidate cytogenetic biomarker with high clinical potential. Additionally, this computational framework could be readily extended to other cancer types, such as leukemia.

## Background

Neuroblastoma (NB) is the most common extracranial solid tumor, usually occurring in early childhood, and it is also the third-most common cancer in babies after leukemia and brain cancer [1]. It is derived from primitive cells of the sympathetic nervous system and usually arises in the abdomen or chest [2]. Approximately 25–50 cases occur per million individuals [3], and 90% of cases arise in children less than 5 years old [4].

To date, two widely used stage classification systems for NB patients have been developed to facilitate clinical research and improve the outcomes of children with NB. The International Neuroblastoma Staging System (INSS), developed in 1988, allows the classification of NB patients into Stage 1, 2A, 2B, 3, 4, and 4S before surgical resection of the tumor [5]. The International Neuroblastoma Risk Group Staging System (INRGSS), proposed in 2009, allows the classification of NB into Stage L1, L2, M and MS based on the results of imaging tests (CT or MRI and MIBG scans) before surgery [6]. To help doctors select the optimal treatment [6], International Neuroblastoma Risk Group (INRG) combines INRGSS information, histologic category, *MYCN* status, and other factors to classify patients into low-, intermediate- and high-risk groups.

Prognostic markers are another important feature used to help predict the patient’s clinical outlook [7–9]. For NB patients, many classic prognostic markers, such as age, tumor histology [10], DNA ploidy [11], transcription instability [12], and *MYCN* amplification [13–15] have been used to predict the prognostic outcome of patients. Among them, *MYCN* is the most critical prognostic marker in NB patients. *MYCN* is a master transcription factor that activates growth-sustaining genes and represses genes that drive differentiation [14]. *MYCN* amplification is found in approximately 25% of all tumors, and most malignant NB patients exhibit *MYCN* amplification [16].

Many studies report the gain of 1q, 2p, and 17q along with a loss of 1p, 3p, 4p, 14q, 11q, 17q, and 19q in the genomes of NB patients (reviewed in [17, 18]). Deletion of chr1p36 occurs in 23–35% of patients [19–21], and deletion of 11q23 occurs in 26–44% of patients [21–23]; each is associated with poor prognosis. The frequent chromosome segment gains and losses [24, 25] but few mutations have been found in NB tumor samples, suggesting that NB is a copy number-driven cancer [17, 26]. Nevertheless, a systematic analysis for the identification of prognostic-associated chromosome bands with frequent gain/loss events for NB patients is still lacking and will be helpful for clinicians in treatment selection.

In this study, we took advantage of the abundant NB genomic data (gene expression data, copy number variation (CNV) data, and clinical information) to systematically identify chromosomal aberration events (gains or losses) that were associated with the clinical outcomes of NB patients. Our analyses revealed a number of chromosomal bands that were frequently amplified or deleted in NB samples with significant associations at the prognostic level. Particularly, some bands (chr11q23, chr11p14) were still predictive of patient survival after adjusting for well-established clinical variables, including *MYCN* amplification status, an extremely widely used prognostic biomarker. These chromosomal aberrations have the potential to be developed into effective cytogenetic markers, as they are visible by microscopic examination. Such a marker can further improve prognostic prediction and patient stratification in NB. Moreover, the computational framework introduced in this article can be readily applied to the identification of cytogenetic markers in other cancer types.

## Methods

### Dataset and data processing

NB gene expression datasets and related clinical data with sufficient overall outcome information were downloaded from the Gene Expression Omnibus (GEO) under accession number GSE62564 (Su et al., *n* = 498). The International Cancer Genome Consortium (ICGC) data portal was accessed under the code NBL-US (Pugh et al., *n* = 249), which contained the segmental chromosome CNV data. The European Bioinformatics Institute was accessed under ID: E-MTAB-179 (Oberthuer et al., *n* = 478). The other dataset used in this study that did not contain survival information was downloaded from the GEO under accession number GSE45478 (Kocak et al., *n* = 123) with the NB segmental CNV information and gene expression profiles. Among them, GSE62564 was generated using the RNA-seq platform, NBL-US and E-MTAB-179 using a one-channel microarray platform, and GSE45478 using a two-channel array platform. A total of 1347 samples were included in these datasets. A summary of these four datasets is provided in Additional file 8: Table S8. The gene expression values obtained from the RNA-seq platform and the one-channel microarray platform were log transformed, and gene-wise mean normalization was performed to obtain the relative expression values for these datasets. The genes associated with positional gene set data were downloaded from the C1 collection of MSigDB (http://software.broadinstitute.org/gsea/msigdb/index.jsp) [27], all bands from the X and Y chromosomes were excluded.

### Mapping the segmental chromosome’s CNV to the chromosome band’s CNV

For each sample, the segmental chromosome’s CNV was mapped to the chromosome band’s CNV. The CNV of chromosome band *i* is defined as follows:

$$ CNV(i)={\sum}_{j=1}^n\frac{l_{ij}}{l_i}\ast SCNV(j), $$

where *SCNV*(*j*) is the CNV value of the *j*th segment chromosome, *l*_*i*_ is the length of chromosome band *i*, *l*_*ij*_ is the length of the overlap between the chromosome band *i* and the segmental chromosome *j*, and *n* is the number of the segmental chromosomes in a given sample.

### Calculation of chromosome band CNV (iCNV) based on the gene expression data

For a given gene expression dataset, all the genes located on a given chromosome band were grouped into a set designated ‘B’, and the rest of the genes located on any other chromosome bands were grouped into a set designated ‘A’. The inferred CNV for a given chromosome band is the value of Student’s *t* statistic comparing the gene sets B and A:

$$ \mathrm{t}=\frac{\overline{x_B}-\overline{x_A}}{\sqrt{\raisebox{1ex}{${s}_B^2$}\!\left/ \!\raisebox{-1ex}{${n}_B$}\right.+\raisebox{1ex}{${s}_A^2$}\!\left/ \!\raisebox{-1ex}{${n}_A$}\right.}} $$

where $\overline{x}$ is the mean, *s*^2^ is the variance, and *n* is the number of genes located in a gene set [28]. For each sample in the dataset, this process was iterated for each chromosome band such that we obtained a matrix of iCNVs for each chromosome band of each sample. If the number of genes located on a chromosome band was less than 10, we considered the Student’s *t* statistic measurement unreliable, and that chromosome band was eliminated from further analysis.

### Survival analysis

A univariate Cox proportional hazards model was fitted to the iCNV for each chromosome band across all samples in a dataset to evaluate the relationship between iCNV and sample survival time. For survival-associated iCNVs of chromosome bands, multivariate Cox proportional hazards models were used to examine prognostic abilities, while potential confounding factors, including *MYCN* status, age, gender, and stage were considered. Kaplan-Meier curves were used to visualize the results from the Cox proportional hazards model. Specifically, the iCNVs were stratified into two groups by the median value for generating the Kaplan-Meier curves.

All survival analyses were conducted in R using the “*survival*” package. Specifically, “*coxph*”, “*survfit*”, and “*surdiff*” were called to create the Cox proportional hazards model, plot Kaplan-Meier curves, and compare the two survival curves.

## Results

### Overview of this study

**Fig. 1:**
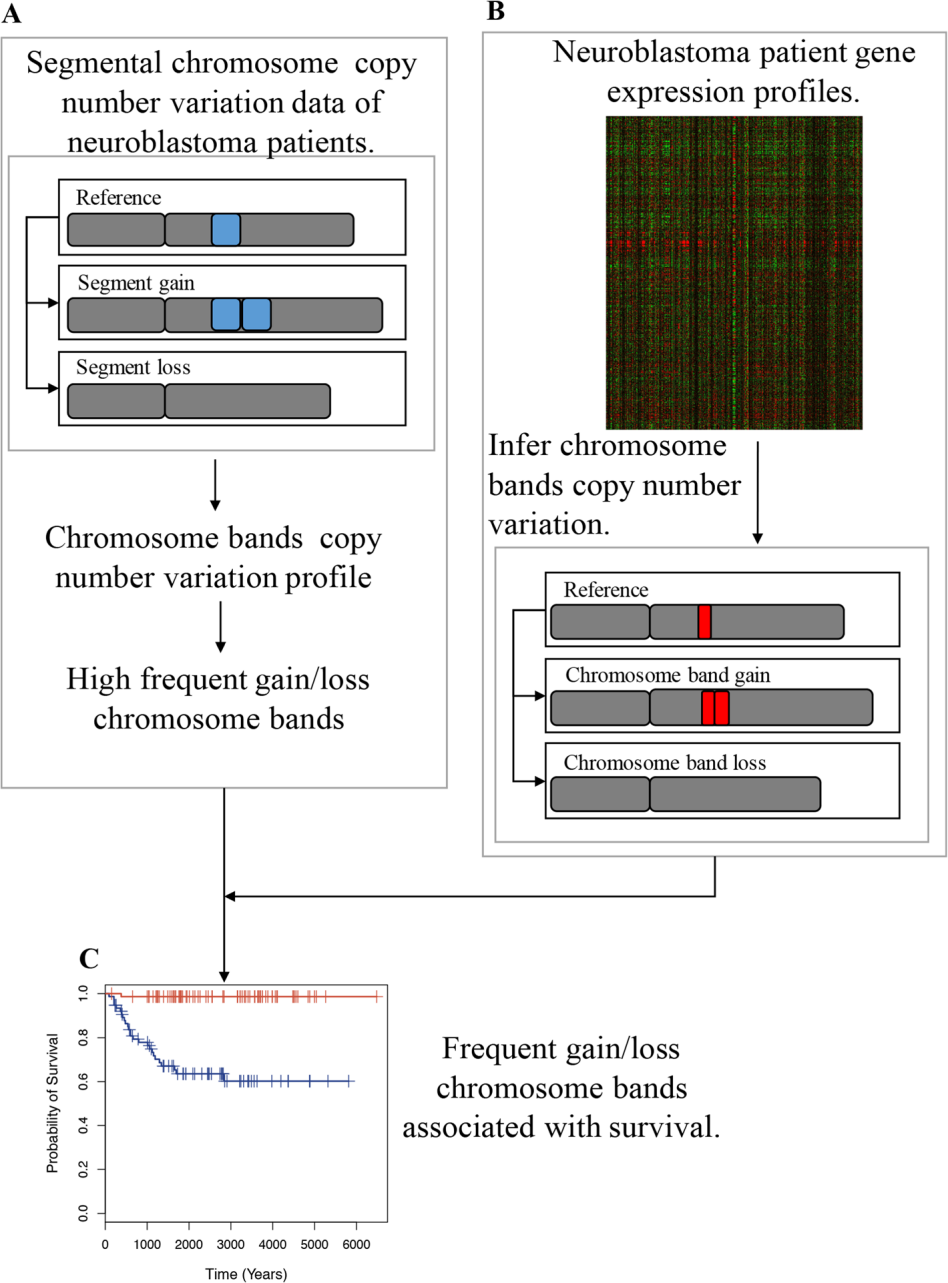
Schematic diagram of our analysis. **a** The segmental chromosome copy number variation data of neuroblastoma patients was used to map to the chromosome band copy number variation, and the frequency of chromosome band gain/loss was obtained. We selected chromosome bands with a gain/loss frequency > 15% as the frequent gain/loss chromosome bands. **b** The neuroblastoma patient gene expression profiles were used to calculate the inferred copy number variation (iCNV) of each chromosome band. **c** A Cox proportional hazard model was used to measure the correlation between frequent gain/loss chromosome band iCNVs and patient survival time

To systematically identify novel candidate cytogenetic biomarkers, we performed an integrative analysis via the following main steps (Fig. 1). First, we analyzed two NB CNV datasets to select chromosomal bands with a high frequency of gain or loss. One of the datasets included samples from all stages [29], while the other included only high-risk NB samples (mostly Stage IV) [30]. By combining results from both datasets, we selected 223 chromosomal bands with > 15% gain/loss frequency in at least one of the datasets. Second, we applied a computational approach to infer sample-specific CNVs for each chromosomal band selected in step 1 based on gene expression data. This approach was applied to Su [31] and Oberthuer [32] NB datasets, which contained both gene expression profiles and clinical information, particularly the survival times of patients. Third, we applied univariate Cox proportional hazards models to examine the association between the resulting inferred copy number values (iCNVs) and patient survival. Finally, we applied multivariate Cox proportional hazards models to select chromosomal bands that remained significantly associated with prognosis after adjusting for critical clinical variables, including age, stage, gender, and *MYCN* amplification status. Of note, we used gene expression data rather than CNV data to determine the association between chromosomal bands and patient survival. We selected gene expression data because more gene expression data are available with a significantly larger sample size and higher quality of survival information than CNV data, thus ensuring sufficient statistical power and rigorous results of our analysis.

### CNV data analysis identified chromosome bands with a high frequency of gain or loss

To identify chromosome bands that were frequently amplified or deleted in NB patients, we analyzed two CNV datasets. Each dataset identified chromosomal segments with abnormal copy numbers determined using array-based comparative genomic hybridization and genome-wide human single nucleotide polymorphism (SNP) arrays 6.0, respectively. One dataset contained 122 samples from different stages (the Kocak dataset) [29], and the other dataset focused on high-risk NB samples, including 149 out of 150 from Stage IV tumors (the Pugh dataset) [30].

**Fig. 2:**
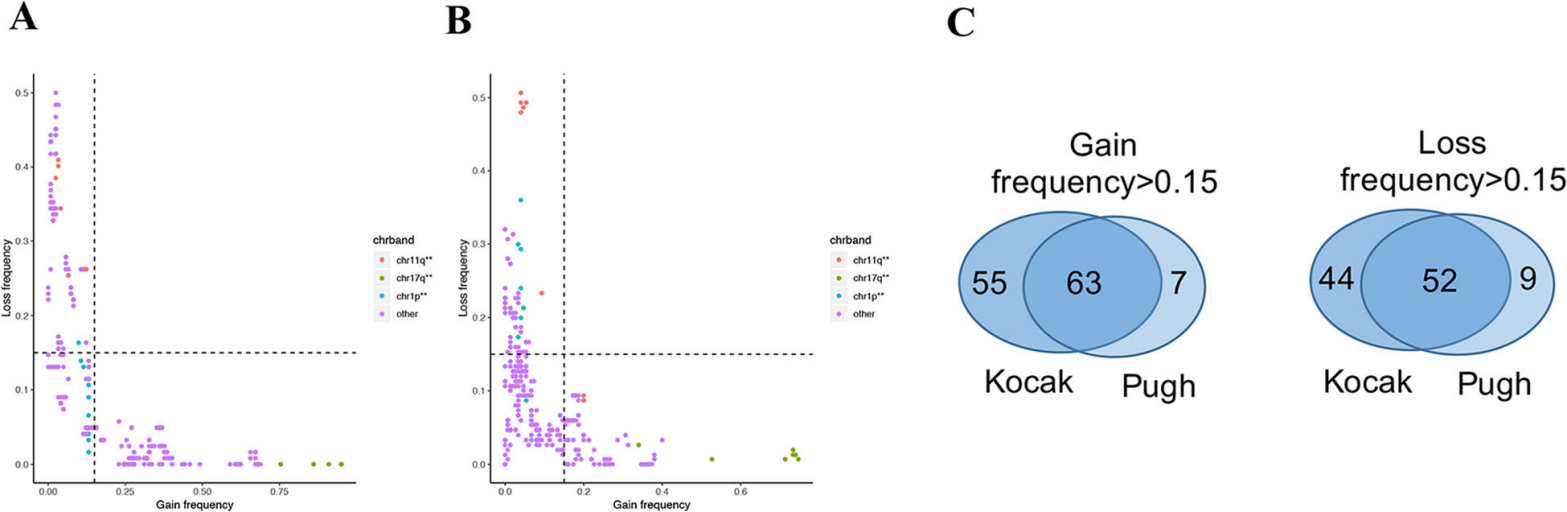
Chromosome band gain/loss frequency in the Kocak and Pugh datasets. **a** The frequency of chromosome band gain/loss on the Kocak dataset, which contains all six stages of neuroblastoma patients. Chromosome bands on the right side of the vertical dashed line or above the horizontal dashed line are considered frequent gain/loss on the Kocak dataset. **b** The chromosome band gain/loss frequency in the Pugh dataset, which contained 1 Stage I patient and 149 Stage IV patients. Chromosome bands on the right side of the vertical dashed line or above the horizontal dashed line were considered to have frequent gain/loss in the Pugh dataset. **c** The number of frequent gain/loss chromosome bands in the Kocak and Pugh datasets. The association of these 223 chromosome bands with frequent gain/loss events with patient survival was studied

By combining the two datasets, our analysis included chromosome bands with high gain/loss frequency generally in all samples and in high-risk samples. Specifically, for each sample, we mapped the aberrant segments to chromosome bands and obtained copy number values of each band (see Additional file 1: Table S1). In Fig. 2a and b, we summarized the frequency of gain/loss for each chromosome band (272 in total excluding the X and Y chromosomes) in the two datasets. As expected, there was a negative correlation between amplification and deletion frequency. Namely, a set of chromosome bands was frequently amplified in NB with high frequency, while a different set of bands was frequently deleted. This enabled us to define frequently amplified and frequently deleted chromosome band sets. By setting the cut-off values to 15%, we identified 118 frequently amplified bands and 96 frequently deleted bands in the Kocak dataset and 70 frequently amplified bands and 61 frequently deleted bands in the Pugh dataset (Fig. 2c).

Although the observed gain/loss frequency was highly consistent, some chromosome bands had notable differences between the two datasets, indicating variations in chromosome aberration events among tumor stages. For example, chr1p36 was lost in 16% of samples in the Kocak dataset but was lost in as high as 36% of Stage IV samples (the Pugh dataset). To obtain a comprehensive list of chromosome bands with high frequency gain/loss events, we took the union of the bands identified from the two datasets, yielding 125 and 105 frequently amplified and frequently deleted chromosome band sets, respectively (Fig. 2c) (Additional file 2: Table S2). Most of the previously established cytogenetic markers are included in our band sets. For example, the well-known chr17q21 [33, 34] was amplified in 95% (the Kocak dataset) and 74% (the Pugh dataset) of samples, while chr11q23 [35, 36] was deleted in 40% (the Kocak dataset) and 49% (the Pugh dataset) of samples.

Interestingly, between the frequently amplified and frequently deleted chromosome band sets, there were 7 overlapping chromosome bands. These 7 chromosome bands were chr11p15, chr11p14, chr11p13, chr11p12, chr11p11, chr11q11, and chr11q12. In the Kocak dataset, they were more likely to be deleted. In the Pugh dataset, they were more likely to be amplified. It is notable that all these chromosome bands are from chromosome 11. Thus, we examined the co-occurrences of the grain/loss events of these bands in samples from the Kocak and Pugh datasets (Additional file 9: Figure S1 and S2). Out of the 60 samples in the Kocak dataset, 34 are associated with whole-chromosome gain (*n* = 3) or loss (*n* = 31) of chromosome 11,45 with whole-arm gain (*n* = 13) or loss (*n* = 32) of chromosome 11p, and 34 with whole-arm gain (n = 3) or loss (n = 31) of chromosome 11q. Out of the 167 samples in the Pugh dataset, 43 are associated with whole-chromosome gain (*n* = 7) or loss (*n* = 36) of chromosome 11, 90 with whole-arm gain (*n* = 48) or loss (*n* = 42) of chromosome 11p, and 45 with whole-arm gain (n = 7) or loss (*n* = 38) of chromosome 11q.

We noted that many of these chromosome bands were consecutive in the genome, e.g., chr1p36, chr1p35, chr1p34, and chr1p33. The chromosome bands from the same clusters were likely associated with the same chromosome gain/loss hot spot. Thus, we produced a list of nonredundant chromosome bands by selecting the most frequently gained or lost bands from each cluster, resulting in a total of 29 frequently amplified and 29 frequently deleted nonredundant bands (Additional file 3: Table S3).

### Copy numbers of chromosome bands can be accurately inferred from gene expression data

To identify cytogenetic events of potential prognostic value, we aimed to examine the association between chromosome band gain/loss events and patient survival in a systematic manner. The above-described Kocak CNV dataset did not provide patient survival information and therefore could not be used for this analysis. The Pugh CNV dataset comprised mostly high-risk patients. However, a few high-quality gene expression datasets from NB patients were generated, which provided expression profiles for a large number of tumor samples and carefully prepared survival information (Additional file 4: Table S4). Therefore, we adapted a previously proposed method [28] to infer the copy number of chromosome bands based on these high-quality gene expression datasets. This method compared the expression of genes located in a chromosome band against other genes and used the Student’s *t* statistic to infer the chromosome band status. This analysis resulted in an inferred copy number value (iCNV) for each chromosome band in each tumor sample.

**Fig. 3:**
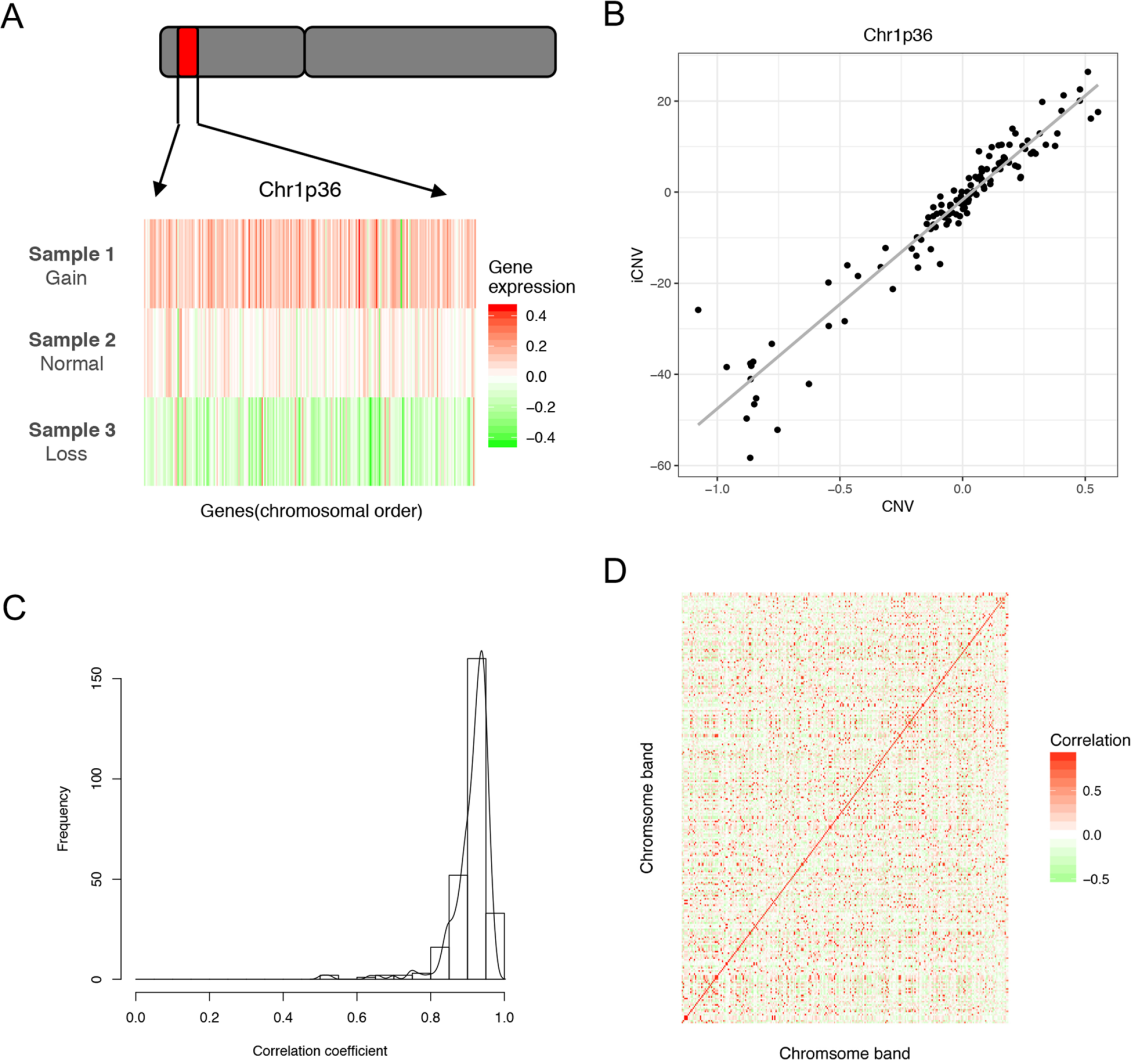
Correlation between chromosome band inferred copy number variation (iCNV) and copy number variation (CNV). **a** The gene expression profile of three patients with gain, normal, and loss of Chr1p36. Genes are sorted according to their position on the chromosome. **b** The correlation between iCNV and CNV of Chr1p36 for all samples. **c** A histogram for the correlation coefficient of chromosome bands across all samples. **d** The correlation matrix heatmap of the chromosome bands across all samples

First, we assessed the performance of this method by comparing the iCNVs to experimentally measured copy number scores using the Kocak dataset. As shown in Fig. 3a, we selected three samples with amplified, normal and deleted chr1p36. The expression of genes in this chromosome band reflected the band status with high fidelity. The iCNVs for this band were also highly correlated (R = 0.97) with the experimentally determined copy numbers (Fig. 3b). We then calculated the correlation coefficients for all of the 272 chromosome bands, and their distribution indicated a high accuracy of the copy number inference (Fig. 3c). More than 80% of chromosome bands had R > 0.8. Moreover, iCNVs for any particular band had the highest correlation with the measured copy number scores of the same band but had much lower correlations with other bands, indicating high specificity (Fig. 3d). Taken together, our results suggested that the iCNV inferred from gene expression data provided an accurate estimation of chromosome band copy numbers.

### Identification of chromosome bands associated with patient prognosis

**Fig. 4:**
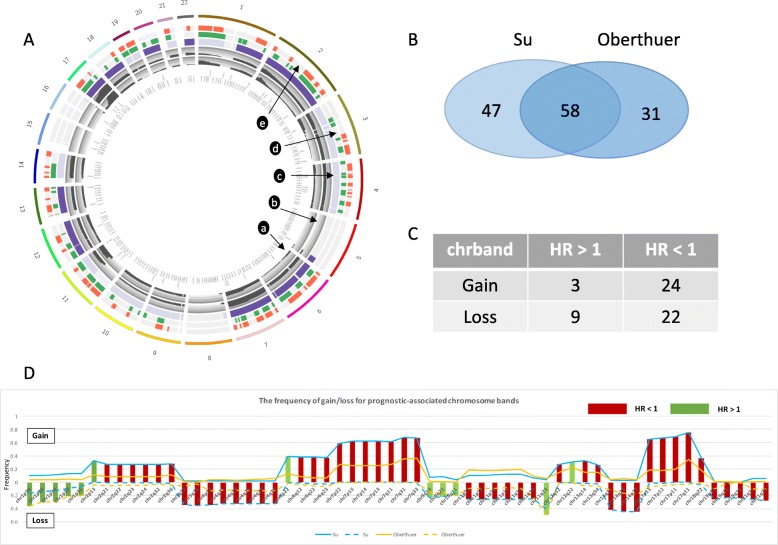
Fifty-eight robust chromosome bands were associated with patient survival. **a** An overview of the 272 chromosome bands with frequency of gain/loss events and prognosis. Track a shows a histogram of the gain/loss frequency in the Pugh dataset. Track b shows a histogram of the gain/loss frequency in the Kocak dataset. Track c shows the gain/loss status for each chromosome band. Dark purple represents frequent gain. Light purple represents frequent loss. Gray represents nonfrequent gain/loss. Track d shows the association between chromosome aberration and prognosis in the Su dataset. Green presents the high frequency of gain/loss chromosome bands associated with prognosis. Gray presents non-association with prognosis. Track e shows the association between chromosome aberration and prognosis in the Oberthuer dataset. Red presents chromosome bands with high gain/loss frequency associated with prognosis. Gray presents non-association with prognosis. **b** The overlapping prognostic-associated chromosome bands in the Su and Oberthuer dataset. **c** The number of frequent gain/loss chromosome bands associated with good/bad outcome. **d** Detailed information on the 58 robust chromosome bands associated with patient survival

**Fig. 5:**
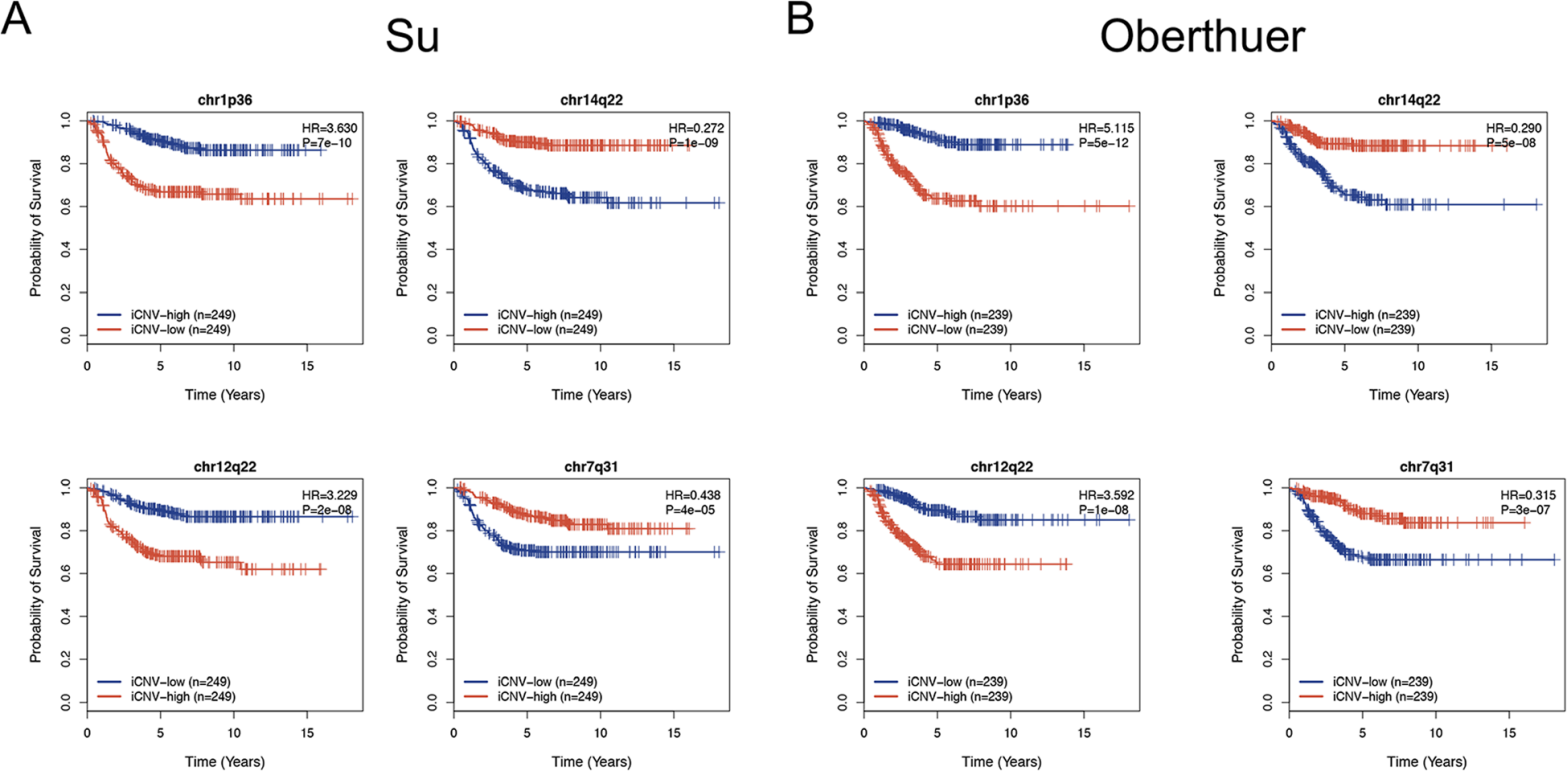
Prediction applications of the iCNV of chromosome bands in the Su and Oberthuer datasets. Loss of chr1p36 and gain of chr12q22 were associated with poor prognosis in both the Su (**a**) and Oberthuer (**b**) datasets. Loss of chr14q22 and gain of chr7q31 were associated with good prognosis in both the Su (**a**) and Oberthuer (**b**) datasets

We then applied the chromosome band copy number inference method to two gene expression datasets, Su [31] and Oberthuer [32], to calculate sample-specific iCNVs. Subsequently, we examined the iCVNs for their association with prognosis. We focused our analysis on the 125 frequently amplified and 105 frequently deleted chromosome bands. We used univariate Cox proportional hazard models to identify chromosome bands that were significantly associated with patient survival. As shown in Fig. 4a, the gain/loss status of the 223 chromosome bands and the prognostic association with patient survival for the two datasets are briefly described. To obtain a list of prognostic chromosome bands of highest confidence, we combined the results from these two datasets and selected the bands that were significant in all datasets (adjusted *p* < 0.05) (Fig. 4b). This combination of results yielded a total of 58 significant chromosome bands (Additional file 5: Table S5). Among the 27 frequently amplified bands, 24 were associated with good survival (HR < 1), and three were associated with poor survival (HR > 1). Among the 31 frequently deleted bands, 22 were associated with good survival (HR < 1), and 9 were associated with poor survival (HR > 1) (Fig. 4c). Detailed information on the 58 prognostic-associated bands is shown in Fig. 4d. Most of the chromosomal bands with aberration events were clearly associated with good prognosis (46/58). Furthermore, we found that most of these bands were either hot spots or near the hot spots, suggesting the importance of each hot spot area in terms of patient prognosis. We further selected four chromosome bands as examples, as shown in Fig. 5a, b. The loss of chr1p36 was associated with poor prognosis in both the Su and Oberthuer datasets, and this finding was consistent with previous reports [37, 38]. We also found that the loss of chr14q22 was associated with good prognosis. The gains of chr12q22 and chr7q31 were associated with poor prognosis and good prognosis, respectively.

It is important to note that the status of *MYCN*, the best-characterized genetic marker associated with poor prognosis in NB [14], may confound our results. To further examine whether the prognostic association of these 58 chromosome bands was independent of this clinical variable, we applied multivariate Cox proportional hazard models to the Su and Oberthuer datasets. The models included sample-specific iCNV for each band, stage, age, gender and status of *MYCN* status as covariates. We selected the chromosome bands with Cox proportional hazards *p*-value < 0.05 in these two datasets which yielded 7 chromosome bands with iCNV associated with patient survival (Additional file 6: Table S6).

**Fig. 6:**
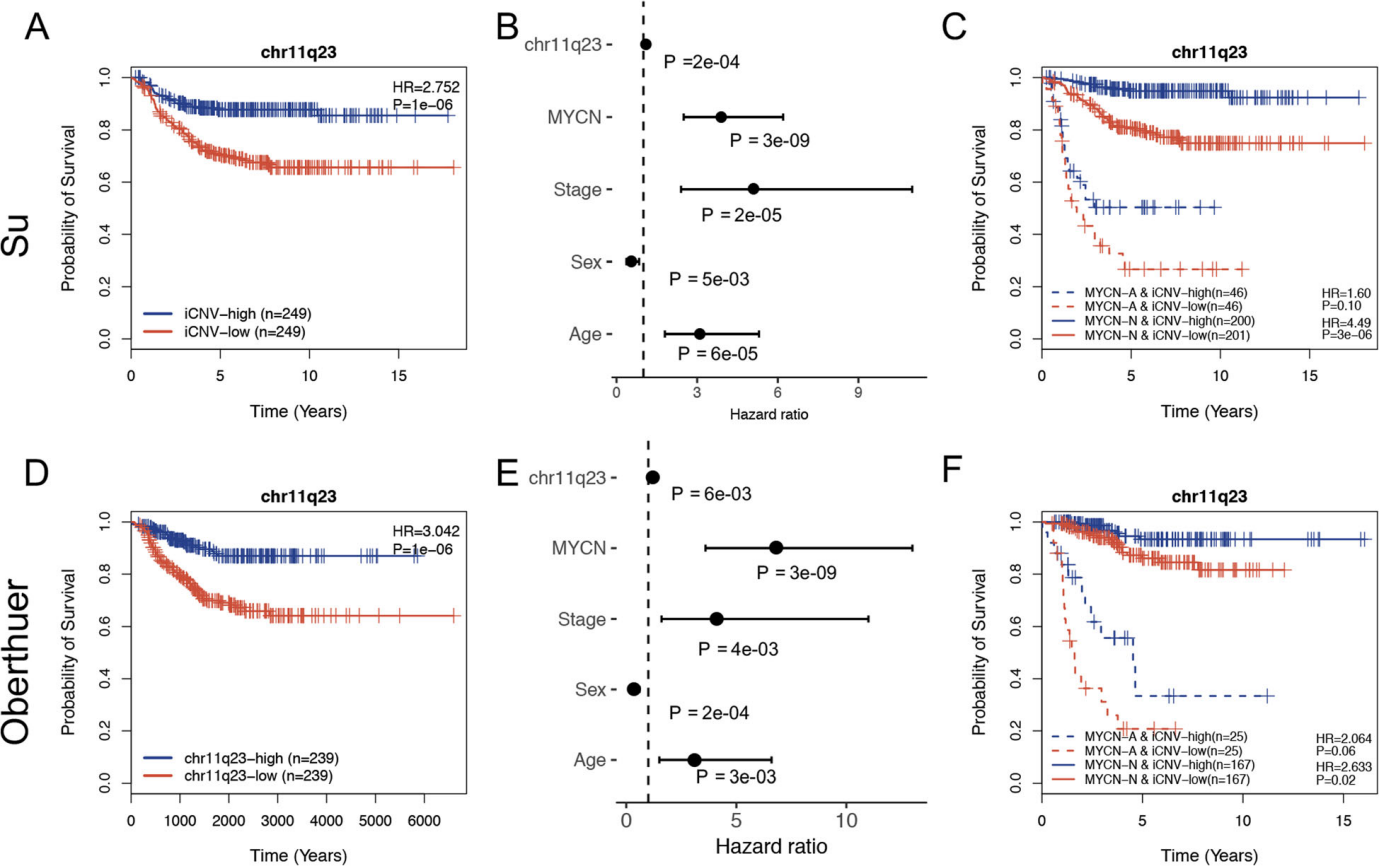
iCNV of chr11q23 as a predictor of survival in Su and Oberthuer datasets. **a**, **d** Loss of chr11q23 was associated with poor outcome in the Su and Oberthuer datasets. **b**, **e** The iCNV of chr11q23 was significantly associated with poor prognosis after taking into account several confounding factors in the two datasets. **c**, **f** The iCNV of chr11q23 was still significant among *MYCN* nonamplification patients and predicted poor prognosis in the Su and Oberthuer datasets

Taking the well-known cytogenetic marker chr11q23 as an example in the Su dataset, loss of chr11q23 was associated with poor prognosis, which was also consistent with a previous report [35] (Fig. 6a). After we considered potential confounding clinical variables including *MYCN* status, stages, sex, and age, using a multivariate Cox proportional hazard model, the iCNV for chr11q23 remained significant (*p* = 2e-04) with a hazard ratio of 1.13 (Fig. 6b). To further examine whether the iCNV for chr11q23 was still significant with patient prognosis in both the *MYCN* amplification patient cohort and the *MYCN* nonamplification patient cohort, we compared the survival curves of four groups stratified based on the level of iCNV and *MYCN* status (Fig. 6c). The iCNV for chr11q23 was still significantly associated with poor prognosis in the *MYCN* nonamplification patient cohort (*p* = 3e-06, HR = 4.49) while was less significant in the *MYCN* amplification patient group. We also performed these analyses on the Oberthuer dataset and obtained similar results (Fig. 6d, e, f).

**Fig. 7:**
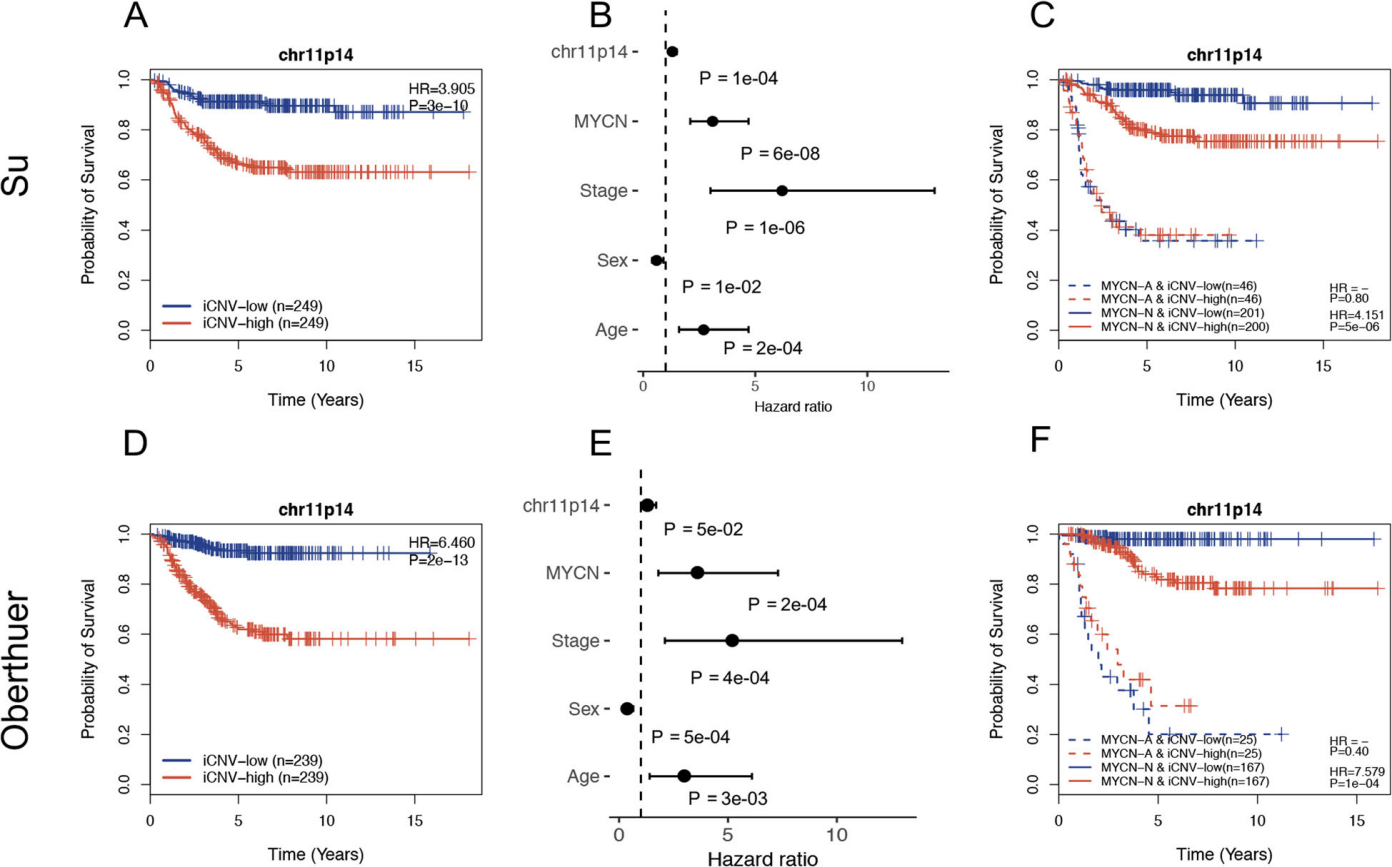
Chr11p14 as a potential cytogenetic marker for predicting survival in the Su and Oberthuer datasets. **a**, **d** Gain of chr11q23 was associated with poor outcome in the two datasets. **b**, **e** The iCNV of chr11p14 was significantly associated with poor prognosis after taking into account several confounding factors in the two datasets. **c**, **f** The iCNV of chr11p14 was still significantly associated with poor prognosis among *MYCN* nonamplification patients in the two datasets

In addition to chr11q23, among the seven chromosome bands, we found that chr11p14 was also a potential novel cytogenetic marker for measuring the progress of NB patients. Chr11p14 was an interesting chromosome band that exhibited a character different from most of the other chromosome bands. It exhibited not only loss events but also gain events. The frequency of gain events in the Pugh dataset was 18%, but the frequency of loss events in the Kocak dataset was 26%. As shown in Fig. 7a, the gain events for chr11p14 were significantly associated with poor prognosis (p = 3e-10, HR = 3.905) in the Su dataset. Furthermore, a significant result was obtained upon applying the multivariate Cox proportional hazard model, as described above (Fig. 7b, p = 1e-04, HR = 1.337). Additionally, among the *MYCN* nonamplification patients, the gain of chr11p14 was significantly associated with poor prognosis (Fig. 7c, p = 5e-06, HR = 4.151). To further validate our results, we performed the same analysis on the Oberthuer dataset and obtained similar results (Fig. 7d, e, f).

The Pugh dataset differs from the two datasets described above; most of the samples in this dataset are Stage IV (216 Stage IV, 1 Stage III and 30 Stage I). To further investigate the association between chromosome band gain/loss and high-risk status for patients, we applied a univariate Cox proportional hazard model and found 10 prognosis-associated chromosome bands (adjust *p* < 0.05) (Additional file 7: Table S7). Seven of the 10 bands were also discovered in the other two datasets (Su and Oberthuer datasets). The other 3 chromosome bands were discovered in either the Su dataset or the Oberthuer dataset.

Taken together, our analysis resulted in a comprehensive list of prognostic-associated chromosome bands. In particular, the chr11p14 gain event provided additional prognostic value in addition to well-established clinical factors, including *MYCN* status, and thereby represents a novel candidate cytogenetic biomarker with high clinical potential.

## Discussion

Chromosomal instability is a hallmark of human cancer and plays an important role during tumorigenesis and progression [39]. Frequent gain or loss of particular chromosome regions has been investigated in certain cancer types, such as leukemia and NB [40, 41]. Some of these recurrent events have been developed into cytogenetic markers to define cancer subtype, predict prognosis, and select effective therapeutic interventions [42]. A list of chromosome aberration events in NB was previously compiled [17]. For example, deletion of chr1p36 [19] and chr11q23 [21] have been reported in 23–35% and 26–44% NB samples, respectively. Both deletions were associated with poor prognosis. Conversely, whole gain of chr17q is associated with good prognosis [34]. Despite the important clinical implications and extensive reports in previous studies, the association of chromosome bands with prognosis has never been investigated in a systematic manner. In this study, we integrated different genomic data with clinical information to systematically identify novel candidate cytogenetic markers for improving NB prognosis.

We utilized two CNV datasets to identify chromosome bands with a high frequency of gain or loss events in NB. This could theoretically also be achieved by examining the iCNV inferred from gene expression data. The iCNV is an essential *t* statistic that compares the relative expression levels of genes in a chromosome band with those not in that chromosome band. As such, each iCNV was associated with a *p*-value that could be estimated by referring to the *t* distributions. Given an NB gene expression dataset, we might calculate sample-specific iCNVs along with corresponding *p*-values and then count the number of samples in which a band shows significantly higher (gain) or lower (loss) iCNV. However, the frequency of a gain/loss event can only be correctly calculated if the relative expression levels of genes were calculated by normalizing against their expression in normal tissue. Unfortunately, no normal control was available in the NB gene expression datasets. Therefore, in our analyses, we used the median expression of genes in all samples to convert absolute gene expression values to relative expression values, which has been proposed in [12]. For this reason, the iCNV calculated from these datasets indicated the copy numbers relative to the median reference rather than a normal control and did not correctly inform the gain/loss frequency of chromosome bands. On the other hand, the iCNV remained effective for examining the association with patient prognosis.

The CNV datasets were not used in our analysis to determine the association of chromosome band gain/loss events with prognosis because of their limitations. The Kocak dataset did not provide survival information, and the Pugh dataset contained only Stage IV NB samples (expect for 1 Stage III sample). In contrast, the gene expression datasets used in our analysis were originally produced for prognostic studies and had large sample sizes. For example, the Su dataset was generated by the SEQC Project, containing 498 carefully selected NB samples with detailed clinical information, including patient survival. In addition, the Su and Oberthuer datasets contained samples from all stages. Combined with the high risk-specific Pugh dataset, these gene expression datasets enabled us to systematically identify chromosome bands that were prognostic in all NB patients or found specifically in high-risk (most Stage IV) patients. Moreover, by combining results from multiple independent datasets, we expected to obtain a list of highly confident prognostic-associated chromosome bands for cytogenetic marker development.

*MYCN* amplification correlates with high-risk disease, has been found in ~ 25% of NB patients, and is widely used as the most critical prognostic marker. For high-risk NB patients, the 5-year survival rate is approximately 40 to 50%. After considering the *MYCN* status, we still found 7 chromosome bands significantly associated with patient survival. Chr11q23 has been well studied. However, gain of chr11p14 was significantly associated with poor prognosis, which has the potential to be a novel cytogenetic biomarker. In the high-risk group (Pugh dataset), we identified 10 chromosome bands associated with patient outcomes that were also found in the other two datasets.

## Conclusions

In conclusion, we performed a systematic analysis that integrated different genomic datasets with clinical information to identify chromosome band gain or loss events associated with NB patient prognosis. Our analysis resulted in a comprehensive list of prognostic chromosome bands supported by strong statistical evidence. In particular, the chr11p14 gain event provided additional prognostic value in addition to well-established clinical factors, including *MYCN* status, and thereby represents a novel candidate cytogenetic biomarker with high clinical potential. Additionally, the computational framework introduced in this article could be readily extended to other cancer types, such as leukemia.

## Supplementary information

**Additional file 1: Table S1.** The copy number values of each chromosomal band on the Kocak dataset and the Pugh dataset.

**Additional file 2: Table S2.** A comprehensive list of chromosome bands with high frequency gain/loss events.

**Additional file 3: Table S3.** A list of nonredundant chromosome bands by selecting the most frequently gained or lost bands from each cluster.

**Additional file 4: Table S4.** The detailed information of 3 NB datasets.

**Additional file 5: Table S5.** The combination of results yielded a total of 58 significant chromosome bands associated with patient prognosis.

**Additional file 6: Table S6.** Seven chromosome bands associated with patient survival after considering the confounding factors.

**Additional file 7: Table S7.** Ten prognosis-associated chromosome bands on the Pugh dataset.

**Additional file 8: Table S8.** Dataset summary.

**Additional file 9: Figure S1.** The landscape of chromosome bands gain/loss on Kocak dataset. **Figure S2.** The landscape of chromosome bands gain/loss on Pugh dataset.

## Data Availability

The datasets analyzed during the current study are available from the Gene Expression Omnibus (GEO) under accession number GSE62564, GSE45478, from The International Cancer Genome Consortium (ICGC) data portal under the code NBL-US, and from The European Bioinformatics Institute under ID: E-MTAB-179. The genes associated with positional gene set data were downloaded from the C1 collection of MSigDB (http://software.broadinstitute.org/gsea/msigdb/index.jsp), all bands from the X and Y chromosomes were excluded.

## Abbreviations

CNV: Copy number variation
GEO: Gene Expression Omnibus
ICGC: International Cancer Genome Consortium
INRG: International Neuroblastoma Risk Group
INRGSS: International Neuroblastoma Risk Group Staging System
INSS: International Neuroblastoma Staging System
NB: Neuroblastoma
SNP: Single nucleotide polymorphism
