# Supplementary material for: Systematic computational identification of prognostic cytogenetic markers in neuroblastoma
Source: BMC Med Genomics. 2019 Dec 12;12:192. doi: 10.1186/s12920-019-0620-6 (PMC6909636; doi:10.1186/s12920-019-0620-6)
Supplement: Supplementary file 9 — Additional file 9: Figure S1. The landscape of chromosome bands gain/loss on Kocak dataset. Figure S2. The landscape of chromosome bands gain/loss on Pugh dataset. [file 12920_2019_620_MOESM9_ESM.docx]

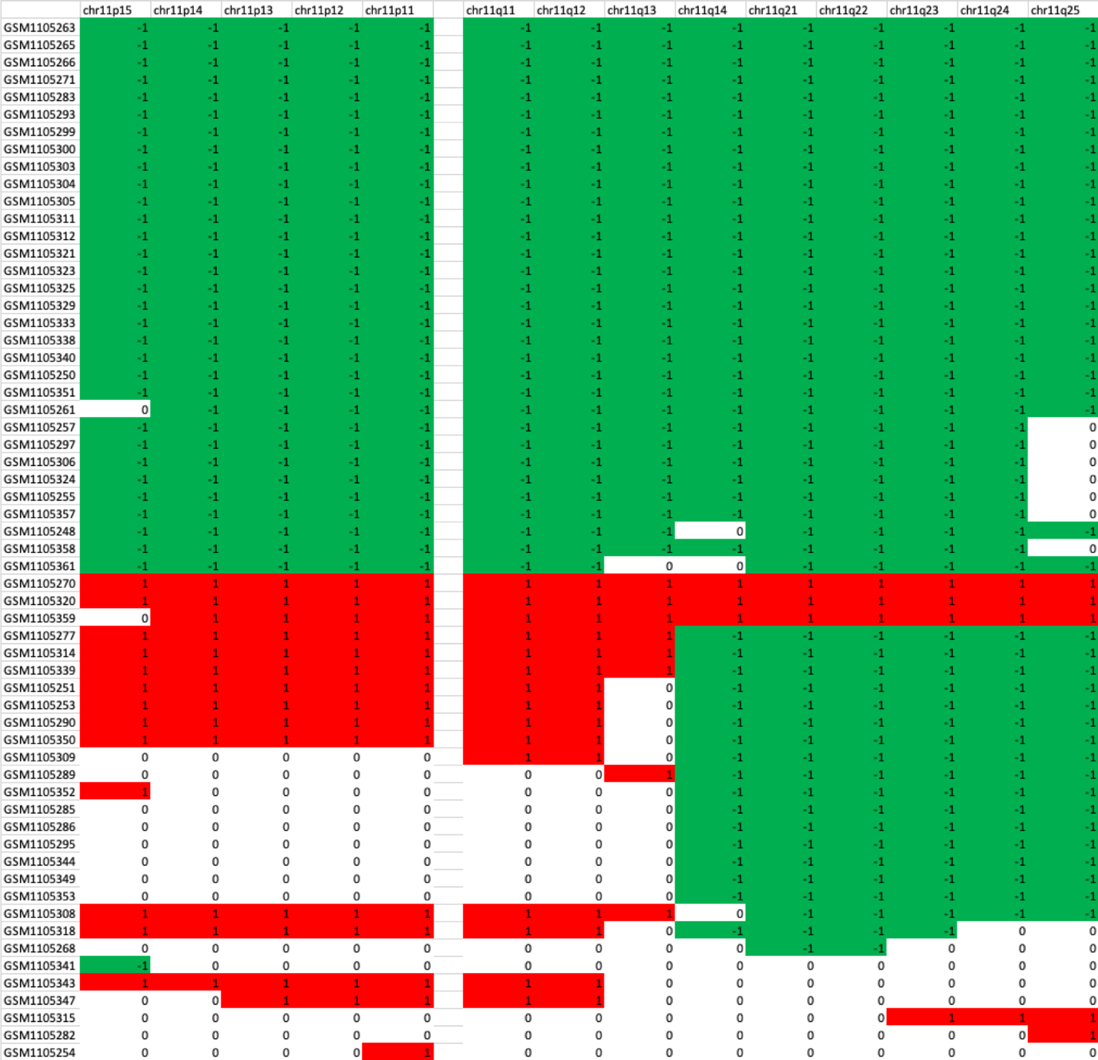


**Figure S1** The landscape of chromosome bands gain/loss on Kocak dataset. The red indicates the gain event, the green indicates the loss event and the white indicates the normal status of chromosome band.


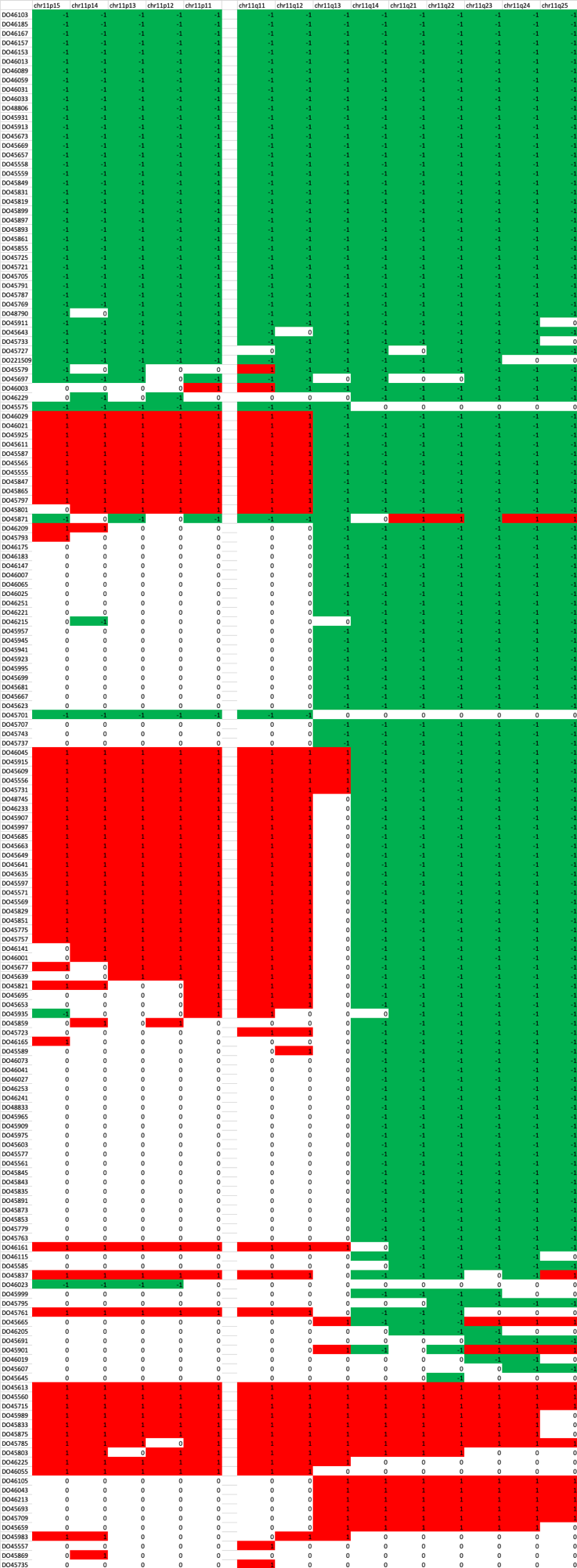


**Figure S2** The landscape of chromosome bands gain/loss on Pugh dataset. The red indicates the gain event, the green indicates the loss event and the white indicates the normal status of chromosome band.
